# Bovine Lactoferrin in the Prevention of Antibiotic-Associated Diarrhea in Children: A Randomized Clinical Trial

**DOI:** 10.3389/fped.2021.675606

**Published:** 2021-06-07

**Authors:** Michal F. Wronowski, Maria Kotowska, Marcin Banasiuk, Artur Kotowski, Weronika Kuzmicka, Piotr Albrecht

**Affiliations:** ^1^Department of Pediatric Gastroenterology and Nutrition, Medical University of Warsaw, Warsaw, Poland; ^2^Department of Pediatrics With Clinical Assessment Unit, Medical University of Warsaw, Warsaw, Poland; ^3^Polish Association for Good Clinical Practice, Warsaw, Poland; ^4^Department of Laboratory Diagnostics and Clinical Immunology of Developmental Age, Medical University of Warsaw, Warsaw, Poland; ^5^Postgraduate School of Molecular Medicine, Medical University of Warsaw, Warsaw, Poland

**Keywords:** lactoferrin, antibiotic-associated diarrhea, children, *Clostridioides difficile*, probiotics

## Abstract

**Introduction:** Antibiotic-associated diarrhea (AAD) is a common adverse reaction to antibiotic treatment affecting up to 21% of children. The aim of the study is to evaluate whether bovine lactoferrin (bLf) might be used for AAD prevention.

**Materials and Methods:** In this prospective, randomized, double-blind, placebo-controlled, single-center study, we enrolled 156 children aged between 1 and 18 years, treated with antibiotic due to acute respiratory or urinary tract infection. We randomly allocated children 1:1 to receive 100 mg of bLf or a placebo twice a day orally for the whole period of antibiotic therapy. The primary outcome was the occurrence of antibiotic-associated diarrhea during and up to 2 weeks after antibiotic therapy. The secondary endpoint was intravenous rehydration or antibiotic withdrawal due to diarrhea. We performed intention-to-treat analysis.

**Results:** We included 150 patients in intention-to-treat analysis. AAD occurred in 16 of 75 (21.3%) patients in bLf group and in 7 of 75 (9.3%) individuals in placebo group [OR = 2.6, (95% CI: 1.01–6.84), *p* = 0.04]. Relative risk was 2.29 (95% CI: 0.89–5.88). The need for intravenous rehydration occurred in one patient in the placebo group (*p* = 0.3). We observed no adverse effects in neither of the groups.

**Discussion:** The trial indicated that bLf is not effective in AAD prevention. The risk for AAD was higher in bovine lactoferrin group as compared with placebo. We registered the study protocol on ClinicalTrials.gov (NCT02626104).

## Introduction

Antibiotic-associated diarrhea (AAD) is a common adverse effect of antibiotics use (1). It may result in antibiotic withdrawal, the need for intravenous rehydration, or hospitalization, complicating the treatment of underlying infection (2). Estimated incidence varies from 11% (3) in an outpatient setting up to 21% (4) among hospitalized children. AAD most frequently occurs 5–10 days after antibiotic treatment initiation but might be observed even up to 8 weeks after the treatment. Several prevention methods have been investigated, but only two probiotic strains, *Lactobacillus rhamnosus* GG and *Saccharomyces boulardii*, are recommended as efficient (5), with the range of risk reduction between 43 and 48% (6, 7). bLf may be another potential preventive option for AAD.

Lactoferrin is a multifunctional glycoprotein, belonging to transferrin family. It is produced through sebaceous secretion in the gastrointestinal, respiratory, and reproductive systems and can be detected in mammalian milk, tears, or semen. Human and bovine lactoferrins are highly homologous (8), showing antimicrobial properties *in vitro*, among other things on AAD etiological factors (9). Lactoferrin is not completely degraded in the human gastrointestinal tract—gastric pepsin cleaves lactoferrin-derived peptides, showing intense bactericidal activity. Bovine lactoferrin was assessed in VLBL neonates for the prevention of late-onset sepsis and necrotizing enterocolitis, showing beneficial effect with no adverse effects (10, 11). The study was an attempt to explore the potential of bLf in the prevention of AAD in children.

## Materials and Methods

### Study Design

We conducted a single-center, randomized, double-blind, placebo-controlled trial in the tertiary Public Pediatric Teaching Hospital, Warsaw, Poland. The trial was registered on ClinicalTrials.gov (NCT02626104). Bioethics Committee of the Medical University of Warsaw accepted the study protocol (KB/103/2015). We run the trial between December 2015 and March 2017.

### Study Population

The inclusion criteria were as follows: (1) children aged 1–18 years; (2) acute respiratory or urinary tract infection, undergoing; (3) antibiotic therapy started up to 24 h before enrollment. Children were excluded if they had severe or invasive bacterial infection, cow's milk allergy, acute or chronic diarrhea, immune disorders, gastrointestinal tract chronic disease, underwent antibiotic therapy during the last 8 weeks, received a probiotic or iron supplementation within 7 days before enrollment, and received antibiotics within 8 weeks before enrollment, of informed consent.

Legal guardian and children >15 years old informed consents were collected.

### Randomization and Blinding

After recruitment, children were randomly allocated to one of two independent groups, assigning consecutive study number. The randomization list was created by an independent statistician, using StatsDirect software, with a block size of 4, allocation 1:1. The allocation was concealed until the recruitment completion, and data analysis was performed. Both participants and investigators were blinded to the assignation. Lactoferrin and placebo were packed in identical sachets containing the powder of the same look and taste.

All the data collected were stored in a locked cabinet.

### Lactoferrin and Placebo Preparations

The lactoferrin and placebo preparations were donated to the Medical University of Warsaw Public Pediatric Teaching Hospital's Pharmacy by Pharmabest, private limited company, Poland. The manufacturer was not involved in the design, conduction, and analysis of this study.

### Primary and Secondary Endpoints

The primary endpoint was the occurrence of antibiotic-associated diarrhea, defined as (1) >3 stools a day or (2) stool consistency change to loose or watery, lasting for at least 2 consecutive days and negative stool testing for rotavirus/adenovirus/norovirus antigen (genotypes GI, GII). The secondary endpoint was intravenous rehydration and/or antibiotic treatment discontinuation due to diarrhea.

### Intervention

We randomly allocated children to the intervention (100 mg lactoferrin + 900 mg maltodextrin twice a day) or placebo group (1,000 mg maltodextrin twice a day) administered orally for the whole period of antibiotic therapy. Parents or legal guardians fulfilled the case report forms indicating the stool number and consistency for whole antibiotic therapy and 14 days after. Consistency was described as follows: diarrhea-like for type 7–6 according to Bristol Stool Chart, normal for 3–5 types and constipated for 1–2.

In case of diarrhea, we ended the intervention and introduced the proper treatment of diarrhea (termination of antibiotic therapy, oral or intravenous rehydration); we took stool samples for culture, glutamate dehydrogenase (GDH), and *Clostridioides difficile* toxins, and ELISA for rotavirus, adenovirus, and norovirus.

If diarrhea occurred in an outpatient setting, legal guardians contacted the investigator (direct contact to investigator available 24/7). We admitted the children to the hospital for clinical assessment, treatment, and stool testing. The investigator performed phone follow-up in three time points—in the middle of the antibiotic treatment, at the end of the antibiotic treatment and, on the 14th day after treatment has ended.

The case report forms in digital or paper were delivered by legal guardians at the end of follow-up or immediately if diarrhea occurred. If no diary has been sent, investigator tried to contact legal guardian every second day for 2 weeks. If failed, patient was described as “lost to follow-up.”

### Sample Size

The main assumption was that bovine lactoferrin would be more effective than probiotics in the AAD prevention. Thus, the clinically significant difference in lactoferrin efficacy would be diarrhea risk reduction by 80%. With parameters α = 0.05, β = 0.1, and enrollment ratio 1:1, this resulted in a sample size of 130 patients. Assuming that a maximum of 20% of the population would be lost to follow-up, the population was enlarged to 156 patients.

### Statistical Methods

Shapiro–Wilk test of normality was used to test distribution of continuous variables. Data were then expressed as median and interquartile range (IQR) and Mann–Whitney *U-*test was used to make comparisons between two groups. Categorical variables were evaluated by χ^2^ or Fisher exact test where appropriate. A *p*-value of <0.05 was considered statistically significant. Data were analyzed using Statistica 12 (Statsoft, Tulsa, Oklahoma, USA).

## Results

**Figure 1:**
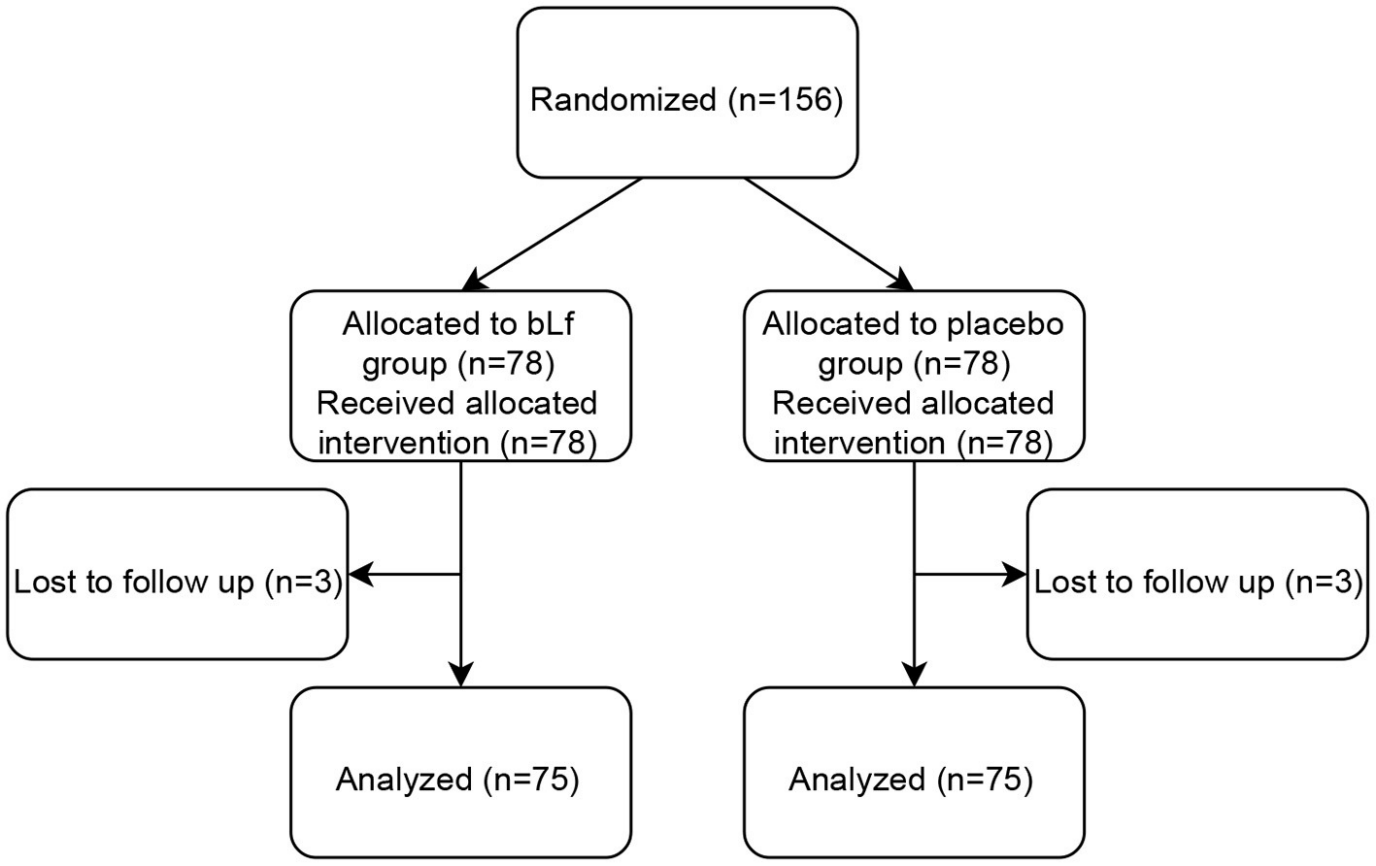
Patient study flow diagram.

Out of 198 patients screened, 34 did not meet the inclusion criteria. In total, 156 children aged 1–16 years were enrolled in the study, 74 boys and 82 girls. Patients were randomized and assigned to either bLf (*n* = 78) or placebo group (*n* = 78). Three individuals in each group were lost to follow-up due to no contact with legal guardians. Thus, 150 children were included in the statistical analysis. Figure 1 shows a flow diagram of subjects' progression in the study.

Baseline demographic and clinical characteristics differed in sex, diagnosis of lower respiratory tract infection, and sub-analysis of antibiotic groups for macrolides, although they were nearly insignificant (Table 1). Diarrhea occurred in 30 of 150 children (20%), and AAD was diagnosed in 23 of them−16 in the bLf group and 7 in the placebo group. Addition of bLf versus placebo to antibiotic therapy increased the risk of AAD [RR 2.29 (95% CI: 0.89–5.88)]. There was no need for antibiotic withdrawal. Intravenous rehydration occurred in one patient in the placebo group (1.3%). The outcome measures are summarized in Table 2. Table 3 shows etiological factors of diarrhea in our patients.

**Table 1:** Baseline characteristics of the study groups.

**Variable**	**bLf group (*n* = 78)**	**Placebo group (*n* = 78)**	***p***
Age, mean	4.28 ± 3.3	4.06 ± 2.6	0.6
Male sex, *n* (%)	30 (38.5)	44 (56.4)	0.03
Diagnosis			
Upper respiratory tract infection, *n* (%)	42 (53.8)	33 (42.3)	0.2
Lower respiratory tract infection, *n* (%)	24 (30.8)	37 (47.4)	0.03
Urine tract infection, *n* (%)	12 (15.4)	8 (10.3)	
Antibiotic			
Penicillins *n* (%)	51 (65.4)	42 (53.8)	0.1
Cephalosporins, *n* (%)	20 (25.6)	21 (26.9)	0.9
Clindamycin, *n* (%)	1 (1.3)	0	1.0
Macrolides, *n* (%)	6 (7.7)	15 (19.2)	0.04
Duration of treatment with antibiotics, mean ± SD days	8.6 ± 2.8	9.2 ± 4.3	0.3

*bLf, bovine lactoferrin*.

**Table 2:** Primary and secondary outcome measures.

**Outcome**	**bLf group (*n* = 75)**	**Placebo group (*n* = 75)**	**Odds ratio (95% CI)**	**Relative risk (95% CI)**
**Primary**, ***n*** **(%)**				
Antibiotic-associated diarrhea	16 (21.3)	7 (9.3)	2.6 (1.02–6.8)	2.29 (0.89–5.88)
**Secondary**, ***n*** **(%)**				
Intravenous rehydration	0	1 (1.28)		
Discontinuation of antibiotic treatment	0	0		

*bLf, bovine lactoferrin*.

**Table 3:** Etiological factor of diarrhea.

**Etiological factor**	**bLf group (*n* = 20)**	**Placebo group (*n* = 10)**
*Clostridioides difficile*	2	0
Rotavirus, adenovirus, and norovirus	4	3
Other pathogens		
*Pseudomonas aeruginosa*	2	0
*Enterobacter cloacae complex* ESBL+	1	1
Non-identified pathogens	11	6

*bLf, bovine lactoferrin; ESBL, extended-spectrum beta-lactamases*.

Both bLf and placebo were well-tolerated; no adverse effects were observed during its administration.

## Discussion

Our study showed that bLf is not effective in the prevention of AAD in children. On the contrary, the risk of diarrhea was higher in bLf group comparing with placebo.

Bovine lactoferrin was previously the object of several randomized clinical trials. bLf alone or in combination with *Lactobacillus rhamnosus* GG reduced the incidence of late-onset sepsis in very low birth weight (VLBW) neonates (10). Also, prophylactic administration of bLf reduced the incidence of invasive fungal infection in VLBL (12). Ochoa et al. run a randomized clinical trial with community-based 6-month supplementation of bovine lactoferrin in Peruvian children showing no decrease in diarrhea incidence, although longitudinal prevalence and severity decreased (13). Other study indicated beneficial effects on respiratory tract infection morbidity in weaned infants (14). However, success in the prevention of one condition does not guarantee therapeutic efficacy in another. To the best of our knowledge, no randomized trials for bLf prevention in AAD in children were published. Studies for AAD prevention mostly evaluate probiotics.

The strengths of this study include the design. This was a randomized controlled trial; participants, researchers, and statistician were blinded. Strict diagnostic definition of diarrhea let us detect the relevant clinical condition. The result of this trial is contrary to expectations and showed a disadvantage of bLf use in the prevention of AAD.

We identified a few limitations in the study. An arguable weakness is the arbitrariness of the bLf dose used in this study—no researchers used bLf in the prevention of AAD in children yet. As reported in previous studies, doses of bLf in various indications ranged widely from 100 to 3,000 mg/day (15).

A potential source of bias for the study was no previous initial microbiological stool testing. The additional altering aspect was the inclusion of both hospitalized and outpatient setting children. We tried to minimize those biases by testing the stool for most common nosocomial infectious agents such as rota/noro/adenoviruses in case of diarrhea (16).

We noted significant differences between study groups. More girls received bLf; however, sex is not a risk factor for AAD (17). Another difference between groups was a higher rate of intravenous antibiotic treatment among hospitalized children; however, the intravenous administration is not significantly predictive of AAD. Also, we found no difference in macrolide usage, aside this is not a high-risk antibiotic group. We used logistic regression in preliminary analysis (Table 4). There was no significant effect of bLf usage on gender, antibiotic preparation, or route given (*p* = 0.071, 0.880, 0.059, and 0.270, respectively). Age at enrollment did have significant effect—older children developed AAD less often (OR = 1.4; 95% CI: 1.07–1.74; *p* = 0.012).

**Table 4:** Logistic regression analysis results in regard to different levels of effect.

**Effect**	**Level of effect**	**Column**	**Estimate**	**SE**	**Wald statistics**	**Lower CL 95.0%**	**Upper CL 95.0%**	**p**	**OR**	**95% CI**	**95% CI**
Intercept		1	2.846	1.334	4.553	0.232	5.460	0.033	17.215	1.261	235.019
bLf usage	1	2	−0.992	0.550	3.253	−2.069	0.086	0.071	0.371	0.126	1.090
Male gender	1	3	0.079	0.521	0.023	−0.942	1.099	0.880	1.082	0.390	3.002
Antibiotic code	1	4	0.080	1.223	0.004	−2.318	2.477	0.948	1.083	0.098	11.910
Antibiotic code	2	5	−1.500	1.150	1.703	−3.754	0.753	0.192	0.223	0.023	2.124
Antibiotic code	3	6	−2.050	1.145	3.202	−4.295	0.195	0.074	0.129	0.014	1.216
Antibiotic code	5	7	−1.190	1.389	0.733	−3.913	1.534	0.392	0.304	0.020	4.635
Mode of antibiotic delivery: 1 = p.o.	1	8	−0.715	0.648	1.217	−1.984	0.555	0.270	0.489	0.137	1.742
Age		9	0.311	0.124	6.262	0.067	0.555	0.012	1.365	1.070	1.742
Scale			1.000	0.000		1.000	1.000				

*Levels of antibiotics are referred to the clarithromycin (level 4).*

*OR, odds ratio; bLf, bovine lactoferrin; CL, confidence level; p.o., per os.*

*Antibiotic codes for levels of effect: 1—amoxycillin, 2—amoxicillin and clavulanic acid, 3—cephalosporins, 4—clarithromycin, and 5—penicillin*.

Several factors may explain the lack of preventive effect of bLf on AAD. bLf shows a direct effect on microbiota and the human gut. bLf has a high affinity for lipopolysaccharide, resulting in common contamination of commercially available lactoferrin preparations; contamination with LPS may influence the results of research (18). On the other hand, another study revealed that purified bLf without LPS might stimulate proinflammatory pathways (19).

In addition, bLf preparations might include iron-depleted apo-Lf or holo-Lf, iron saturated. The bacteriostatic effect is a feature of apo-bLf that deprives iron from the environment limiting bacterial cell growth (20). However, growth-limiting properties of holo-bLf might be iron independent as showed in the human gut model of *Clostridioides difficile* infection, in which holo-bLf showed limitation of *Clostridioides difficile* growth and toxin production (21).

Exclusion criterion in our study was a diagnosis of allergy to cow's milk. We considered bLf strong allergenicity as the dose used in this study was multiply higher than average consumption of cow's milk containing 31.78–485.63 μg/ml of bLf (22). Brock showed that bLf is highly antigenic in healthy infants after exposure to high levels to the protein. Incubation of holo-Lf with anti-lactoferrin IgG found in this population resulted in the liberation of free iron. Antibodies to bLf are detected in a substantial part of the population (23).

Another bias altering the study outcome is the natural history of infections such as otitis media or pneumococcal pneumonia that may manifest with diarrhea (24, 25).

The statistical analysis was completed and the results obtained, we made a decision to perform further testing of bLf product used in the study. The cultures of the preparations were negative. The manufacturer provided a certificate that preparation is LPS-free. Department of Laboratory Diagnostics and Clinical Immunology of Developmental Age run a test showing that the preparation used in this trial inhibits neutrophil extracellular traps (NETs). NETs are structures composed of nuclear chromatin and bactericidal proteins that disarm and kill bacteria extracellularly (26). Previous studies suggested that lactoferrin serves as an intrinsic inhibitor of NETs release into the circulation (27). Another study theorizes NETs may function as “a safety net” confining the proinflammatory neutrophil granule proteins (28).

Contrary to expectations, this study did not find bLf to be prevention for AAD in children. The findings of this research are important because previous research showed no serious adverse effects related to bLf. The mechanism of diarrhea remains unclear. We consider that a combination of bLf and antibiotics may trigger the diarrhea. However, this result has not been described; therefore, it needs to be interpreted with caution.

## Data Availability Statement

The raw data supporting the conclusions of this article will be made available by the authors, without undue reservation.

## Ethics Statement

The studies involving human participants were reviewed and approved by Ethics Committee at Medical University of Warsaw. Written informed consent to participate in this study was provided by the participants' legal guardian/next of kin.

## Author Contributions

MW and MK: conceptualization and investigation. MW, MK, AK, and WK: methodology. MB: data curation. PA: supervision. MW: writing of the manuscript. MW, MK, AK, and PA: editing and revision of the manuscript. All authors contributed to the article and approved the submitted version.

## Conflict of Interest

The lactoferrin and placebo preparations were donated to the Medical University of Warsaw Public Pediatric Teaching Hospital's Pharmacy by Pharmabest, private limited company, Poland). The manufacturer was not involved in the design of the study, collection, analysis and interpretation of data, the writing of the report and decision to submit this manuscript for publication. The authors declare that the research was conducted in the absence of any commercial or financial relationships that could be construed as a potential conflict of interest.
